# Near-Adult Height Outcomes in Patients Treated With rhIGF-1 for Severe Growth Failure: Real-World IGFD Registry Data

**DOI:** 10.1210/clinem/dgaf390

**Published:** 2025-07-08

**Authors:** Marta Ramon-Krauel, Michel Polak, Mohamad Maghnie, Joachim Woelfle, Caroline Sert, Valérie Perrot, Peter Bang

**Affiliations:** Hospital Sant Joan de Déu, Institut de Recerca Sant Joan de Déu, 08950 Barcelona, Spain; Centro de Investigación Biomédica en Red de Diabetes y Enfermedades Metabólicas Asociadas, Instituto de Salud Carlos III, 28029 Madrid, Spain; Endo-ERN European Reference Network on Rare Endocrine Conditions, 1105 AZ Amsterdam, The Netherlands; Department of Pediatric Endocrinology, Gynaecology, and Diabetology, Assistance Publique—Hôpitaux de Paris, Hôpital Universitaire Necker-Enfants Malades, IMAGINE Institute, INSERM U1016, Université Paris Cité, 75015 Paris, France; Endo-ERN European Reference Network on Rare Endocrine Conditions, 1105 AZ Amsterdam, The Netherlands; Department of Pediatrics, IRCCS Istituto Giannina Gaslini, 16100 Genova, Italy; Department of Neuroscience, Rehabilitation, Ophthalmology, Genetics, Maternal and Child Health, University of Genova, 16132 Genova, Italy; Department of Pediatrics and Adolescent Medicine, Friedrich-Alexander-University Erlangen-Nürnberg, 91054 Erlangen, Germany; Ipsen, 92100 Boulogne-Billancourt, France; Ipsen, 92100 Boulogne-Billancourt, France; Division of Pediatrics, Department of Biomedical and Clinical Sciences, Faculty of Health Sciences, Linköping University, 581 83 Linköping, Sweden

**Keywords:** rare growth disorder, severe primary insulin-like growth factor-I deficiency, adult height, global IGFD registry

## Abstract

**Context:**

The Global Increlex^®^ Growth Forum Database (IGFD) Registry monitors real-world effectiveness and safety of recombinant human IGF-1 (rhIGF-1; Increlex^®^ [mecasermin]) treatment in children and adolescents with severe growth failure due to severe primary IGF-I deficiency (SPIGFD).

**Objective:**

To report characteristics, effectiveness, and safety data from patients receiving rhIGF-1 treatment who achieved near-adult height (NAH), and determine factors that predict height gain to NAH.

**Methods:**

Descriptive analyses of patients included in the Global IGFD Registry (NCT00903110) who achieved NAH are reported for the overall population, treatment-naïve prepubertal (NPP) patients, and patients with Laron syndrome. Linear regression analyses of height gain to NAH are also reported.

**Results:**

One hundred and two patients enrolled in the Global IGFD Registry achieved NAH at data cut-off (April 20, 2023). Mean age at rhIGF-1 treatment initiation was 11.8 years; median treatment duration was 3.9 years. Mean (SD) height SD score (HtSDS) gain from rhIGF-1 initiation to NAH was 0.9 (1.1). In NPP patients, mean (SD) HtSDS gain was 1.4 (1.0). Almost half of NPP patients reached NAH within the normal range. Despite improved height in patients with Laron syndrome, only 10.5% reached NAH within the normal range; 3 patients with Laron syndrome were NPP. Treatment naivety was predictive of height gain in the overall NAH population. Safety data aligned with previous reports.

**Conclusion:**

In a real-world setting, despite patients with SPIGFD initiating rhIGF-1 treatment at a relatively advanced age, rhIGF-1 treatment resulted in improved NAH. The greatest improvements in height outcomes were observed in NPP patients.

**Trial registration:**

NCT00903110.

Severe primary IGF-I deficiency (SPIGFD) is a rare growth disorder defined by extreme short stature (height standard deviation score [HtSDS] ≤ −3), deficiency in serum levels of IGF-I (below the 2.5th percentile for age and sex in the European Union and IGF-I SD score [SDS] ≤ −3 in the United States), and normal or elevated levels of GH, indicating GH insensitivity (1). SPIGFD is associated with neonatal hypoglycemia that persists throughout childhood and postnatal growth failure in early childhood, leading to extreme proportionate short stature and persisting to adulthood if untreated (2-4). In addition, patients with SPIGFD have midfacial hypoplasia with frontal bossing, small genitalia, delayed puberty, a high-pitched voice, truncal obesity, underdeveloped muscles, and thin bones (2, 4). These abnormalities, in particular short stature, have been shown to impact psychological and social well-being in children and adults (5).

The most severely affected individuals with SPIGFD are those with Laron syndrome; HtSDS for untreated patients with Laron syndrome ranges from −4 to −10 (6). Laron syndrome was first described as being associated with a pathological autosomal recessive GH receptor (GHR) gene variant; other genetic causes of SPIGFD have since been described (2, 7, 8). The global prevalence of SPIGFD is unclear, due in part to inconsistencies in SPIGFD diagnoses based on national growth charts that vary between countries and commercially available IGF-I assays that lack sensitivity and consistency (1, 9). However, SPIGFD is considered to be a rare disease (1, 10). As such, there is limited awareness and understanding of this disease among healthcare professionals, which poses significant challenges to the diagnosis and treatment of those affected (9).

Mecasermin (Increlex^®^) is a DNA-derived recombinant human IGF-1 (rhIGF-1). rhIGF-1 therapy was approved by the United States Food and Drug Administration in 2005 and the European Medicines Agency in 2007 for the treatment of growth failure in children and adolescents aged 2 to 18 years with confirmed SPIGFD (11, 12). The goal of rhIGF-1 treatment is to allow patients to reach an improved adult height (AH), while considering the therapy's risk-benefit profile (11, 12). There are currently no other approved treatments in this indication (10).

Previous reports in small cohorts of patients have indicated that patients treated with rhIGF-1 have improved AH, with the greatest gain in HtSDS occurring in the first year of rhIGF-1 treatment and first-year height velocity ranging from 6.9 cm/year to 9.3 cm/year (13-16). A study of 21 patients followed to near AH (NAH) reported an improvement in height velocity from 3.1 cm/year in untreated patients to 7.4 cm/year during the first year of rhIGF-1 treatment and a HtSDS gain at NAH of 1.9 (13).

Treatment-naïve patients who initiate treatment at the prepubertal stage have been reported to display more rapid height gain than patients previously exposed to growth-promoting therapies or initiating treatment during puberty (15, 17). SPIGFD delays puberty, and a previous study reported that this delay remains despite rhIGF-1 treatment (18). However, these patients have an apparent normal pubertal height gain (18). Whether initiating rhIGF-1 treatment during puberty can lead to a near-normal pubertal height gain was not addressed.

The Global Increlex^®^ Growth Forum Database (IGFD) Registry was established to collect long-term effectiveness and safety data on the use of rhIGF-1 therapy in children with growth failure due to SPIGFD in real-world clinical practice (19). To our knowledge, data on a large cohort of patients with SPIGFD treated with rhIGF-1 who have achieved NAH in a real-world setting are lacking.

The objective of the present analyses was to describe the characteristics, effectiveness, and safety data in a larger cohort of patients receiving rhIGF-1 therapy and achieving NAH in the real-world setting and to determine the factors that predict HtSDS gain at NAH.

## Methods

### Study Design

The Global IGFD Registry is an ongoing, descriptive, multicenter, prospective, open-ended, noninterventional, postauthorization surveillance registry (NCT00903110) (15, 19). The registry has collected real-world clinical data on the use of rhIGF-1 from 10 European countries since December 2008 and from 8 European countries and the United States since 2021 (data from the United States were not included in this study as no patients had reached NAH by the cut-off date). One aim of the Global IGFD Registry is to follow patients for at least 5 years after the end of rhIGF-1 treatment. Children are eligible to participate in the global registry if they are initiating treatment with rhIGF-1 or were previously treated with rhIGF-1 by a qualified practitioner and are deemed to have SPIGFD as per the approved rhIGF-1 label (11, 12). It is important to note that the inclusion criteria in the European IGFD (Eu-IGFD) Registry, an earlier version of the Global IGFD Registry, were broader, and all patients aged between 2 and 18 years with growth failure, with or without SPIGFD, and initiating or receiving rhIGF-1 therapy could be included (15). As the Global IGFD Registry is an extension of the Eu-IGFD Registry, some patients included in the current analyses were not reported to have SPIGFD. Children entering the registry are required to give informed consent, where appropriate, in addition to mandatory consent from their parents or legally authorized representatives. Children participating in a clinical trial for short stature or rhIGF-1, with any contraindication to rhIGF-1 as per the approved label, or with a medical history of neoplasia or closed epiphyses are excluded. The Global IGFD Registry is conducted in compliance with independent ethics committees/institutional review boards, informed consent regulations, the Declaration of Helsinki, the International Conference on Harmonization, and Good Epidemiological Practice Guidelines.

For the purpose of this study, patients were followed until they had reached NAH (defined as last height velocity <1 cm/year, or with final AH ticked as Yes in the electronic case report form, or with “Attained AH” as reason for end of treatment or end of registry). If a patient had not reached NAH at the end of treatment or after 5-years posttreatment, the follow-up period was extended.

Patients enrolled in the Global IGFD Registry who had achieved NAH at the cut-off date (April 20, 2023) were included in these analyses. Data were collected using an electronic case report form, which included clinical data from patients' medical records and comprised baseline characteristics; treatment outcomes; auxological parameters, including HtSDS at baseline and NAH; and treatment-emergent adverse events (TEAEs). The overall NAH population comprised all patients with complete clinical data at baseline who had received at least 1 dose of rhIGF-1 and patients who stopped treatment (or discontinued the registry) and had been followed up until they had reached NAH. All patients had attended at least 1 follow-up visit.

### Statistical Analysis

Descriptive analyses were conducted on patients who achieved NAH in the overall population in those who were treatment-naïve and received their first rhIGF-1 dose at the prepubertal stage (naïve prepubertal [NPP]), and in those with Laron syndrome. Evolution over time of the main effectiveness endpoints, ie, HtSDS change from baseline (ΔHtSDS) and height velocity, as well as HtSDS and HtSDS gain from baseline to NAH are reported. HtSDS was calculated as previously described (15). Univariate and multivariate linear regression analyses were conducted to identify factors that predict HtSDS gain from rhIGF-1 initiation to NAH in the overall and NPP populations. The following factors were included in the univariate analysis: sex, age at treatment initiation, biological mother height, biological father height, baseline HtSDS, presence of Laron syndrome (defined by a pathological GHR defect), predicted AH (PAH; as reported by the investigator and calculated based on various methods, most frequently the Tanner Whitehouse and Bayley-Pinneau methods) (20, 21), mean dose of rhIGF-1, SPIGFD diagnosis, prepubertal during first year of treatment, treatment duration, and (for overall population only) patients naïve from growth-promoting treatments (recombinant human GH [rhGH], rhIGF-1, and steroids). Estimates were calculated with 95% confidence intervals for each factor, and factors with *P*-values <.2 were retained for multivariate analyses.

## Results

### Patient Characteristics

**Figure 1.**
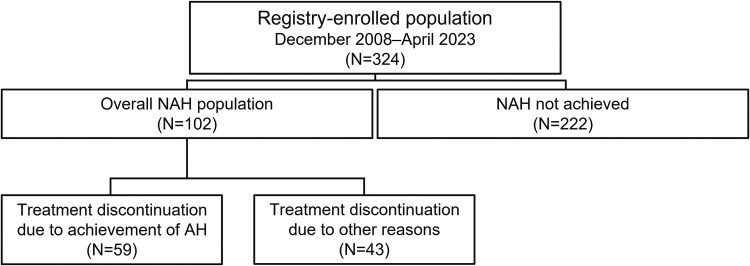
Patient disposition. The overall NAH population included all patients who had reached NAH at data cut-off (April 20, 2023). Over half of these patients discontinued treatment because they had reached AH; the remaining patients, who discontinued treatment for other reasons, such as lack of effectiveness or shortage of rhIGF-1, were followed until they reached NAH after the end of treatment. Abbreviations: AH, adult height; NAH, near-adult height.

At data cut-off (April 20, 2023), 102 out of 324 patients enrolled in the Global IGFD Registry had reached NAH (Fig. 1). Over half of patients (n = 59) discontinued treatment because they had reached AH (Table 1, Fig. 1); the remaining 43 patients, who discontinued treatment for other reasons such as lack of effectiveness or shortage of rhIGF-1, were followed until they reached NAH after end of treatment.

**Table 1.** Clinical and demographic characteristics of patients achieving NAH

Characteristic	Overall (n = 102)	NPP*^a^* (n = 45)	Laron syndrome (n = 19)
Boy, n (%)	67 (65.7)	26 (57.8)	10 (52.6)
SPIGFD, n (%)	87 (85.3)	40 (88.9)	19 (100.0)
NPP, n (%)	45 (44.1)	45 (100.0)	3 (15.8)
Treatment-naïve, n/N (%)	58/102 (56.9)	45/45 (100.0)	5/19 (26.3)
At least 1 genetic test performed*^b^*			
n/N (%)	58/99 (58.6)	20/43 (46.5)	19/19 (100.0)
GH receptor deletion/defect			
n tested	29	5	19
Present, n (%)	19 (65.5)	3 (60.0)	19 (100.0)
Age at rhIGF-1 initiation, years			
Total			
n	102	45	19
Mean (SD)	11.8 (3.4)	10.1 (3.3)	12.6 (4.5)
Boys			
n	67	26	10
Mean (SD)	12.6 (3.1)	10.8 (3.3)	15.0 (2.6)
Girls			
n	35	19	9
Mean (SD)	10.3 (3.4)	9.2 (3.1)	9.9 (4.7)
Age at rhIGF-1 end, years			
Total			
n	102	45	19
Mean (SD)	16.1 (2.7)	15.4 (2.8)	17.9 (2.5)
Boys			
n	67	26	10
Mean (SD)	16.8 (2.4)	16.4 (2.3)	19.4 (1.7)
Girls			
n	35	19	9
Mean (SD)	14.8 (2.7)	14.0 (3.0)	16.2 (2.1)
Pubertal stage at rhIGF-1 initiation, n (%)			
Total			
n	99	45	17
Stage 1	69 (69.7)	45 (100.0)	10 (58.8)
Stage 2	14 (14.1)	0	2 (11.8)
Stage 3	12 (12.1)	0	3 (17.6)
Stage 4	3 (3.0)	0	2 (11.8)
Stage 5	1 (1.0)	0	0
Missing	3	0	2
Boys			
n	64	26	8
Stage 1	42 (65.6)	26 (100.0)	4 (50.0)
Stage 2	10 (15.6)	0	1 (12.5)
Stage 3	11 (17.2)	0	3 (37.5)
Stage 4	0	0	0
Stage 5	1 (1.6)	0	0
Missing	3	0	2
Girls			
n	35	19	9
Stage 1	27 (77.1)	19 (100.0)	6 (66.7)
Stage 2	4 (11.4)	0	1 (11.1)
Stage 3	1 (2.9)	0	0
Stage 4	3 (8.6)	0	2 (22.2)
Stage 5	0	0	0
Missing	0	0	0
Bone age at baseline, year			
n	18	7	3
Mean (SD)	10.8 (2.7)	8.9 (2.6)	11.2 (2.8)
Bone age at rhIGF-1 end, years			
n	78	32	15
Mean (SD)	13.7 (2.6)	12.9 (2.9)	14.3 (2.9)
Difference in chronological age – bone age at baseline, years			
n	18	7	3
Mean (SD)	2.3 (1.4)	2.4 (1.1)	3.1 (0.9)
Difference in chronological age – bone age at end of rhIGF-1 treatment, years			
n	83	37	15
Mean (SD)	1.6 (1.7)	1.5 (1.7)	2.2 (1.8)
BMI at baseline, SDS			
n	85	37	18
Mean (SD)	−0.7 (1.6)	−1.1 (1.4)	0.8 (1.5)
BMI at last measure on rhIGF-1 treatment, SDS			
n	102	45	19
Mean (SD)	−0.2 (1.5)	−0.4 (1.4)	1.1 (1.2)
BMI at NAH, SDS			
n	101	45	19
Mean (SD)	−0.2 (1.6)	−0.4 (1.5)	1.0 (1.3)
Total rhIGF-1 treatment duration, years			
n	102	45	19
Median	3.9	4.7	4.6
Q1; Q3	2.3; 6.0	3.5; 7.0	2.1; 7.3
Treatment duration during the prepubertal period, years			
n	69	45	10
Median	2.4	2.4	2.9
Q1; Q3	1.4; 4.0	1.3; 3.6	2.0; 5.1
Treatment duration during puberty, years			
n	97	44	17
Median	2.7	2.8	2.9
Q1; Q3	1.4; 3.7	1.3; 3.7	2.0; 4.2
At least 1 concomitant treatment with GnRH,			
n (%)	6 (5.9)	1 (2.2)	3 (15.8)
Main reason for treatment discontinuation, n (%)			
n	99	45	19
Attained AH*^c^*	59 (59.6)	27 (60.0)	13 (68.4)
AE	3 (3.0)	1 (2.2)	2 (10.5)
Noncompliance	3 (3.0)	0	0
Lack of effectiveness	13 (13.1)	7 (15.6)	1 (5.3)
Shortage of rhIGF-1	6 (6.1)	5 (11.1)	0
Non-physician decision*^d^*	3 (3.0)	2 (4.4)	0
Physician decision	1 (1.1)	0	0
Other	11 (11.1)	3 (6.7)	3 (15.8)

NAH population.

Abbreviations: AE, adverse event; AH, adult height; BMI, body mass index; NAH, final adult height; NPP, naïve prepubertal; rhIGF-1, recombinant human IGF-1; SDS, SD score; SPIGFD, severe primary IGF-I deficiency; Q1/Q3, lower/upper quartile.

^*a*^Tanner stage 1 of genital development for boys and breast development for girls, respectively.

^*b*^Genetic tests were performed for various genetic defects and polymorphisms.

^*c*^Some patients were followed until they attained AH but remained on treatment.

^*d*^Patient/parent/legally authorized representative decision.

The clinical and demographic characteristics of patients reaching NAH are presented in Table 1. In the overall NAH population, 85.3% (n = 87/102) were diagnosed with SPIGFD, 44.1% were NPP (n = 45/102), and 18.6% (n = 19/102) presented with Laron syndrome. Three patients with Laron syndrome were NPP. In total, 58.6% (n = 58/99) of patients underwent any genetic testing. Of the patients tested, 25.0% (n = 2/8) of those tested for a GH gene defect were positive, 65.5% (n = 19/29) tested for a GHR gene defect were positive, 28.6% (n = 2/7) of those tested for an IGF-I gene defect were positive, and 25.0% (n = 1/4) of those tested for a PTPN11 gene defect were positive. There were no patients with STAT5b (n = 0/3), SHOX (n = 0/10), ALS (n = 0/7), or PAPP2A (n = 0/0) gene defects reported among patients reaching NAH. Of patients with Laron syndrome, 15.8% received at least 1 concomitant dose of gonadotropin-releasing hormone (GnRH) agonist; 5.9% and 2.2% of patients in the overall and NPP populations received at least 1 concomitant dose of GnRH agonist treatment.

At rhIGF-1 treatment initiation, 69.7% (n = 69/99) of patients in the overall NAH population were prepubertal (Tanner stage 1). Mean (SD) ages at treatment initiation and treatment end were 12.6 (3.1) years and 16.8 (2.4) years for boys and 10.3 (3.4) years and 14.8 (2.7) years for girls, respectively. Patients in the NPP population were slightly younger at treatment initiation and treatment end than the overall NAH population, while patients with Laron syndrome were slightly older than the overall population. Patients in the overall NAH population had a median (Q1; Q3) treatment duration of 3.9 (2.3; 6.0) years. Median treatment duration was almost 1 year longer in the NPP and Laron syndrome populations than in the overall NAH population. For NPP patients, median (Q1; Q3) treatment duration in the prepubertal period was 2.4 (1.3; 3.6) years and during puberty was 2.8 (1.3; 3.7) years.

Patients in the overall NAH population were older at treatment initiation than patients in the Global IGFD Registry enrolled population (mean [SD]: 11.8 [3.4] vs 9.4 [4.1] years) (Table 1, Supplementary Table 1 (22)); this was also observed in the NPP and Laron syndrome populations.

### Effectiveness of rhIGF-1 in Patients Who Reached NAH

Mean (SD) HtSDS gain from rhIGF-1 initiation to NAH was 1.4 (1.0) in the NPP population and mean (SD) difference between NAH and PAH was −3.6 (12.8) cm (Table 2). Of the NPP patients, 54.3% (n = 19/35) were responders, defined as a HtSDS change of ≥0.3 at year 1, and 46.7% (n = 21/45) of NPP patients were in the normal AH range (HtSDS > −2) at NAH (Table 2). Of the remaining 16 NPP patients considered nonresponders at year 1, 6 (37.5%) were in the normal AH range. NPP patients who started rhIGF-1 treatment at ≤10 years of age had a numerically greater ΔHtSDS at each year of treatment than patients starting rhIGF-1 treatment at >10 years of age, while mean height velocity was similar between these groups (Supplementary Fig. 1 (22)).

**Table 2.** Auxological parameters in patients achieving NAH

	Overall (n = 102)	NPP*^a^* (n = 45)	Laron syndrome (n = 19)
Height at rhIGF-1 initiation, cm			
Total			
n	92	41	18
Mean (SD)	124.9 (18.0)	118.6 (19.2)	119.8 (25.6)
Boys			
n	59	23	10
Mean (SD)	129.4 (16.1)	122.7 (17.2)	133.3 (18.7)
Girls			
n	33	18	8
Mean (SD)	116.9 (18.8)	113.3 (20.7)	102.9 (23.6)
HtSDS at rhIGF-1 initiation			
n	92	41	18
Mean (SD)	−3.6 (1.4)	−3.6 (1.4)	−5.0 (1.8)
95% CI	−3.9; −3.4	−4.0; −3.1	−5.9; −4.2
PAH, cm			
Total			
n	65	29	12
Mean (SD)	159.8 (13.7)	159.3 (12.1)	154.1 (18.6)
Boys			
n	38	13	5
Mean (SD)	163.5 (13.1)	162.3 (13.4)	158.6 (23.4)
Girls			
n	27	16	7
Mean (SD)	154.7 (13.0)	156.8 (10.7)	151.0 (15.6)
Mid-parental target height, cm			
Total			
n	91	42	17
Mean (SD)	166.3 (9.6)	165.3 (9.3)	165.1 (9.8)
Boys			
n	58	25	9
Mean (SD)	171.2 (7.5)	171.3 (5.4)	171.8 (8.1)
Girls			
n	33	17	8
Mean (SD)	157.7 (6.2)	156.4 (6.4)	157.5 (4.9)
NAH, cm			
Total			
n	102	45	19
Mean (SD)	153.4 (13.4)	155.9 (13.7)	144.2 (14.0)
Boys			
n	67	26	10
Mean (SD)	157.5 (12.6)	161.3 (14.1)	152.6 (11.7)
Girls			
n	35	19	9
Mean (SD)	145.5 (11.2)	148.5 (9.0)	134.9 (10.3)
HtSDS at NAH			
n	102	45	19
Mean (SD)	−2.8 (1.7)	−2.2 (1.4)	−4.1 (1.9)
95% CI	−3.1; −2.5	−2.6; −1.8	−5.0; −3.2
SDS >−2, n (%)	34 (33.3)	21 (46.7)	2 (10.5)
Difference between NAH HtSDS and HtSDS at rhIGF-1 initiation*^b^*			
n	92	41	18
Mean (SD)	0.9 (1.1)	1.4 (1.0)	1.0 (1.1)
95% CI	0.7; 1.1	1.1; 1.7	0.4; 1.6
Difference between NAH and PAH, cm*^b^*			
n	65	29	12
Mean (SD)	−6.9 (11.9)	−3.6 (12.8)	−16.7 (15.9)
95% CI	−9.8; −3.9	−8.4; 1.3	−26.9; −6.6
Difference between NAH and mid-parental target height, cm*^b^*			
n	91	42	17
Mean (SD)	−12.8 (13.2)	−8.7 (11.9)	−20.3 (11.4)
95% CI	−15.5; −10.1	−12.4; −4.9	−26.1; −14.4
HtSDS change ≥0.3 at year 1			
n	79	35	16
Yes, n (%)	41 (51.9)	19 (54.3)	10 (62.5)

NAH population.

Abbreviations: CI, confidence interval; HtSDS, height SD score; NAH, near-adult height; NPP, naïve prepubertal; PAH, predicted adult height; rhIGF-1, recombinant human IGF-1; SDS, SD score.

^*a*^Tanner stage 1 of genital development for boys and breast development for girls, respectively.

^*b*^Difference calculated only for patients with data available for both parameters.

In the Laron syndrome population, HtSDS gain from treatment initiation to NAH was lower than the NPP population (mean [SD]: 1.0 [1.1]), although there was a large variation in response in the Laron syndrome group (Table 2). Furthermore, only 3 patients were NPP and 14 patients were receiving prior growth-promoting therapies (4 patients received GH; 11 patients received IGF-1). Data from the 3 NPP patients with Laron syndrome demonstrated that rhIGF-1 treatment initiated between 2 and 8 years of age resulted in a total HtSDS gain ranging from 1.4 to 3.3 from the start of treatment to NAH, with the highest gain in height observed in the shortest patient at rhIGF-1 initiation. Patients with Laron syndrome were also seemingly shortest at NAH (mean [SD] HtSDS: −4.1 [1.9]), and only 10.5% of these patients were in the normal AH range at NAH (Table 2). However, patients with Laron syndrome appeared to have the lowest HtSDS at baseline, lowest NAH, and largest difference between NAH and PAH (mean [SD]: −5.0 [1.8]; 144.2 [14.0] cm; −16.7 [15.9] cm, respectively).

Mean (SD) HtSDS gain from rhIGF-1 initiation to NAH in the overall NAH population was 0.9 (1.1), lower than in the NPP population, and only 33.3% of patients achieved a height in the normal adult range at NAH (Table 2). The mean (SD) NAH HtSDS for the overall NAH population was −2.8 (1.7), and the mean (SD) difference between NAH and PAH was −6.9 (11.9) cm.

**Figure 2.**
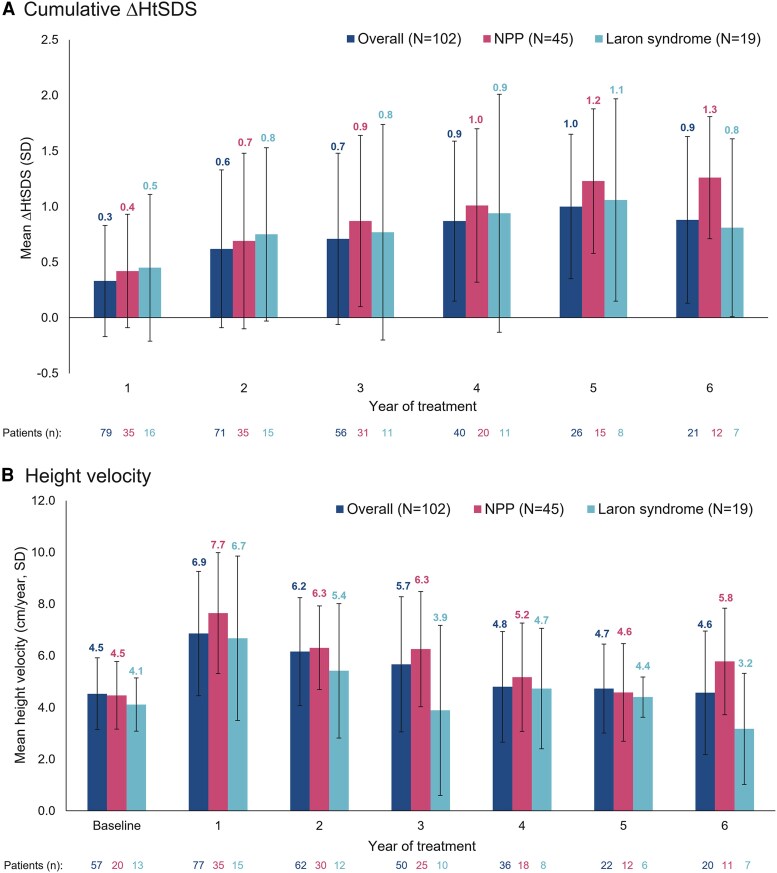
Cumulative ΔHtSDS (A) and annualized height velocity (B) in patients reaching NAH. Cumulative ΔHtSDS and annualized height velocity were analyzed in the overall NAH population, NPP population, and Laron syndrome population. Error bars represent SD. Abbreviations: HtSDS, height SD score; NAH, near-adult height; NPP, naïve prepubertal; rhIGF-1, recombinant human IGF-1; ΔHtSDS, HtSDS change from baseline.

Mean cumulative ΔHtSDS increased over time in all populations, with the greatest height velocity observed in the first year of treatment (Fig. 2). Mean height velocity reduced each year but remained above baseline through 5 years of follow-up. Mean HtSDS at NAH and mean difference between NAH HtSDS and HtSDS at rhIGF-1 initiation were similar irrespective of age at treatment initiation in NPP patients (≤10 or >10 years of age; Supplementary Table 2 (22)).

Auxological parameters for patients in the Global IGFD Registry overall and in the NPP and Laron syndrome populations are shown in Supplementary Table 3 (22).

Body mass index (BMI) increased moderately from treatment initiation to last recorded measure on rhIGF-1 treatment for all subgroups; mean BMI SDS at last recorded measure on rhIGF-1 treatment was the same or comparable to mean BMI SDS at NAH for all populations (Table 1). BMI SDS changes from treatment initiation to NAH were similar across populations.

### Predictors of HtSDS Gain to NAH

In the NPP population, HtSDS gain from rhIGF-1 initiation to NAH was greater in patients with lower baseline HtSDS (*P* < .001), higher PAH (*P* = .028), and greater biological mother height (*P* = .039) (Table 3). HtSDS gain at year 1 of rhIGF-1 treatment was not a predictor of HtSDS gain from rhIGF-1 initiation to NAH in NPP patients (Table 3). Being naïve to all treatments (*P* < .001) and having a greater biological mother height (*P* = .029) were predictive of HtSDS gain to NAH in the overall NAH population (Table 4).

**Table 3.** Univariate and multivariate linear regression analyses for HtSDS gain to NAH in the NPP population achieving NAH (n = 45)

Explanatory variable	n	Estimate (95% CI)	*P*-value
Univariate analyses			
Sex (girl)	41	0.15 (−.50; .80)	.643
Age at rhIGF-1 initiation by 1 unit increment, years	41	−0.01 (−.11; .08)	.757
BMH by 1 unit increment, cm	38	0.05 (.01; .08)	.007
BFH by 1 unit increment, cm	38	0.02 (−.03; .07)	.367
HtSDS at baseline	41	−0.25 (−.47; −.04)	.021
Laron syndrome	41	1.17 (−.01; 2.34)	.052
PAH by 1 unit increment, cm	27	0.03 (−.00; .06)	.052
Mean dose of rhIGF-1 by 1 unit increment, µg/kg BID	41	−0.01 (−.03; .00)	.139
SPIGFD	41	0.54 (−.43; 1.51)	.268
Prepubertal patients during the first year of rhIGF-1 treatment	41	0.32 (−.45; 1.10)	.401
rhIGF-1 treatment duration by 1 unit increment, days	41	0.00 (−.00; .00)	.507
HtSDS change at year 1 ≥ 0.3 SD	35	0.41 (−.20; 1.02)	.177
Multivariate analysis			
HtSDS at baseline by 1 unit increment	26*^a^*	−0.45 (−.66; −.23)	<.001
Predicted adult height by 1 unit increment, cm	26*^a^*	0.03 (.00; .06)	.028
BMH by 1 unit increment, cm	26*^a^*	0.04 (.00; .07)	.039

Height velocity at year 1 not tested in the univariate analysis because there were more than 10% missing data. Covariates used in the multivariate regression were HtSDS at baseline, mean dose of rhIGF-1 over the course of the study (µg/kg BID), BMH (cm), PAH (cm). Presence of Laron syndrome is not included in the multivariate analysis due to its correlation with HtSDS (*P* < .001).

Abbreviations: BFH, biological father height; BID, twice daily; BMH, biological mother height; CI, confidence interval; HtSDS, height SD score; NAH, near-adult height; NPP, naïve prepubertal; rhIGF-1, recombinant human IGF-1; SDS, SD score; SPIGFD, severe primary IGF-I deficiency.

^*a*^19 patients in the NPP population were not included in the multivariate analysis as they were missing data from ≥1 covariate.

**Table 4.** Univariate and multivariate linear regression analyses for HtSDS gain to NAH in the overall NAH population (n = 102)

Explanatory variable	n	Estimate (95% CI)	*P*-value
Univariate analyses			
Sex (girl)	92	0.23 (−.24; .71)	.335
Age at rhIGF-1 initiation by 1 unit increment, years	92	−0.05 (−.12; .01)	.107
BMH by 1 unit increment, cm	84	0.03 (.00; .05)	.042
BFH by 1 unit increment, cm	85	0.03 (−.00; .06)	.059
HtSDS at baseline	92	−0.07 (−.23; .10)	.427
Laron syndrome	92	0.09 (−.49; .67)	.753
PAH by 1 unit increment, cm	61	0.03 (.01; .04)	.009
Mean dose of rhIGF-1 by 1 unit increment, µg/kg BID	92	−0.00 (−.01; .01)	.966
SPIGFD	92	0.20 (−.44; .84)	.531
Prepubertal patients during the first year of rhIGF-1 treatment	89	0.59 (.14; 1.4)	.011
Patients naïve from all treatments (rhGH, rhIGF-1, steroids)	92	0.85 (.42; 1.28)	<.001
rhIGF-1 treatment duration by 1 unit increment, days	92	0.00 (−.00; .00)	.092
Multivariate analysis			
Patients naïve from all treatments (rhGH, rhIGF-1, steroids)	84	0.78 (.37; 1.18)	<.001
BMH by 1 unit increment, cm	84	0.03 (.00; .05)	.029

Height velocity at year 1 not tested in the univariate analysis because there were more than 10% missing data. Covariates used in the multivariate regression were treatment duration (days), age at first rhIGF-1 intake (years), BMH (cm), BFH (cm), PAH (cm), prepubertal patients during the first year of treatment, patients naïve from all treatments (rhGH, rhIGF-1, steroids).

Abbreviations: BID, twice daily; BFH, biological father height; BMH, biological mother height; CI, confidence interval; NAH, near-adult height; NPP, naïve prepubertal; rhIGF-1, recombinant human IGF-1; SDS, SD score; SPIGFD, severe primary IGF-I deficiency.

### Safety in the Overall NAH Population

**Figure 3.**
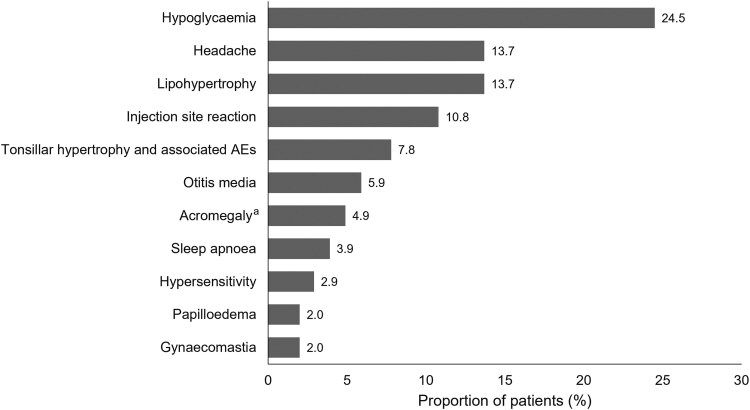
Targeted TEAEs in patients achieving NAH. The proportion of patients reporting targeted TEAEs in the overall NAH population (n = 102) is reported. ^a^Acromegalic facial changes were coded as acromegaly. Abbreviations: AE, adverse event; NAH, near-adult height; TEAE, treatment-emergent AE.

The proportions of patients with TEAEs are shown in Table 5. In total, 65.7% (n = 67/102) of patients reported at least 1 TEAE and 15.7% (n = 16/102) of patients reported at least 1 serious TEAE; 53.9% (n = 55/102) of patients reported at least 1 treatment-related TEAE. Three patients experienced a benign neoplasia (treatment-emergent adverse event of special interest); 1 had dysplastic naevus and 2 had melanocytic naevus. All 3 cases were deemed nonserious and related to treatment by the investigator. Hypoglycemia was the most frequent targeted TEAE (Fig. 3). The number of hypoglycemic events per patient-year was highest in year 1 (0.249) of rhIGF-1 treatment, decreased in year 2 (0.033), and remained ≤0.130 to year 6 (Supplementary Table 4 (22)). Data on rhIGF-1 exposure for the NAH population were not available. However, based on the overall safety population (n = 322) exposure of 1297 patient-years, exposure for the 102 patients in the NAH population was estimated to be 398 person-years. From the inception of the Global IGFD Registry in December 2008 to the cut-off date of April 2023, there were a total of 22 serious hypoglycemia events reported in the overall safety population. Among these, 9 serious AEs involving hypoglycemia requiring hospitalization were documented, with no reports of hypoglycemia-associated seizures during this period. In 2024, a single case of seizure attributed to hypoglycemia was reported in 1 patient enrolled in the registry; the event resolved without sequelae.

**Table 5.** Proportion of patients with TEAEs in the overall NAH population (n = 102)

	n (%)	Total number of TEAEs
At least 1 TEAE	67 (65.7)	250
At least 1 treatment-emergent AESI (neoplasia)	3 (2.9)	3*^a^*
At least 1 serious TEAE	16 (15.7)	24
At least 1 related TEAE	55 (53.9)	155
At least 1 TEAE leading to treatment withdrawal	3 (2.9)	3
At least 1 TEAE leading to death	0	0

Abbreviations: AESI, adverse event of special interest; NAH, near-adult height; TEAE, treatment-emergent adverse event.

^*a*^All reported AESIs were neoplasia: 1 dysplastic naevus and 2 melanocytic naevus, all deemed nonserious and related to treatment by the investigator.

## Discussion

Among patients enrolled in the Global IGFD Registry, 102 had reached NAH by April 2023 and were included in the present study. To our knowledge, the Global IGFD Registry comprises the largest cohort of patients achieving NAH during or after rhIGF-1 treatment in the real-world setting.

Over half of patients who achieved NAH had at least 1 genetic test performed, with approximately two-thirds of patients tested for GHR gene defects in the overall and NPP populations reporting a GHR deletion or gene defect. Whether these were homozygous or compound heterozygous pathological genetic alterations cannot be confirmed from registry data. Most patients with SPIGFD in the Global IGFD Registry lack a reported genetic cause; however, a systematic whole genome sequencing approach has not been conducted in many of these patients (17).

Effectiveness and safety data in this analysis aligned with results observed in previous studies of patients with severe growth failure treated by rhIGF-1 therapy and achieving AH and other clinical and observational study data from smaller numbers of patients (13-16). Previously, Backeljauw and colleagues studied a smaller cohort of 21 patients with SPIGFD achieving NAH and observed HtSDS gain from rhIGF-1 initiation to NAH was higher than in the present study (1.9 vs 0.9), as was year 1 height velocity (7.4 cm/year vs 6.9 cm/year) (13). However, patients from the Backeljauw et al study were all naïve to IGF-1 therapy; started treatment at a younger age; had a lower HtSDS and height velocity at baseline, suggesting a more severe phenotype; and had a higher proportion of patients with GHR defects, indicating the presence of Laron syndrome, than patients achieving NAH in the Global IGFD Registry (13). As such, there was a higher proportion of patients with more severe disease and treatment-naïve patients in the NAH cohort from the Backeljauw et al study, which may explain the improved rhIGF-1 effectiveness they reported, compared with the overall Global IGFD Registry NAH population. When examining NPP patients who achieved NAH in the Global IGFD Registry, HtSDS gain to NAH and year 1 height velocity data were more closely aligned to the data from the Backeljauw et al cohort (13) in naïve patients. However, both the overall and NPP populations in the present study had higher proportions of patients who attained a normal height range (33.3% and 46.7%, respectively) than the previous smaller NAH cohort, where only 14.3% of patients reached a normal AH range (13). Slightly more than half of the NPP patients in the present study were responders to treatment (HtSDS change of ≥0.3 at year 1), in accordance with our previous report (17). Examining the total HtSDS gain to NAH specifically in this responder group may further indicate the potential effectiveness of rhIGF-1 treatment. The results from the present study nevertheless confirmed previous clinical trial data suggesting that early treatment with rhIGF-1 in patients naïve to previous hormonal growth-promoting therapy improves height gain to AH more effectively than treatment at a later pubertal stage (13) and demonstrated that these results are applicable in a real-world setting.

Patients with Laron syndrome in this analysis had a lower baseline HtSDS compared with the overall and NPP populations, in line with previous studies (15, 17), and achieved a lower NAH. NAH for untreated patients with Laron syndrome ranges from 100 to 136 cm in females and 115 to 142 cm in males, with HtSDS ranging from −4 to −10 (6, 23). In the present study, patients with Laron syndrome treated with rhIGF-1 attained a mean (SD) HtSDS at NAH of −4.1 (1.9), suggesting an improved AH compared with patients who do not receive treatment (3). However, only 10.5% of patients with Laron syndrome reached a height within the normal adult range. It is important to note that only 5 of the 19 patients with Laron syndrome were treatment-naïve at rhIGF-1 initiation. The remaining patients had been treated with growth-promoting therapies prior to starting rhIGF-1 treatment, and therefore their HtSDS at rhIGF-1 initiation may not accurately reflect their baseline HtSDS prior to any growth-promoting treatment. It is therefore not possible to fully evaluate rhIGF-1 effectiveness in this group as their HtSDS gain following rhIGF-1 treatment may be influenced by their height gain while receiving growth-promoting therapy. In this analysis, less than 10% of all patients received concomitant GnRH treatment, though it is noted that in all populations the number of patients with this combination was low and likely did not impact the overall results. In previous studies of rhIGF-1 treatment, there was no difference in AH between patients with or patients without prior GnRH analogue exposure (13, 14). Finally, although data from the 3 NPP patients with Laron syndrome could not be presented for confidentiality reasons, we observed that HtSDS improvement was greatest in this population, in line with previous reports (17).

Annualized ΔHtSDS and height velocity in the overall population and NPP population were similar to those reported in previous cohorts of patients with IGF-I deficiency with long-term rhIGF-1 treatment (13, 14). Height velocity was highest in the first year of treatment, in line with the previous studies (13, 14). Data collected from the Eu-IGFD Registry up to May 2017 demonstrated that rhIGF-1 treatment effectiveness varied between subgroups (15, 17). In the present analysis, the first year annualized ΔHtSDS and height velocity in the overall NAH population did not differ from NPP patients, most likely due to the contribution of prepubertal patients in the overall population. Prepubertal patients on rhIGF-1 treatment often have delayed puberty yet have a peak pubertal height velocity of 7.9 cm/year in boys and 6.8 cm/year in girls (18), similar to the first-year height velocity in the overall NAH population and NPP patients in the present study. However, the greater HtSDS gain from rhIGF-1 initiation to NAH in the NPP population compared with the overall NAH population is indicative of the potential effectiveness of rhIGF-1 on NAH in the NPP group.

In the current study, it was observed that NAH was generally lower than PAH. This discrepancy highlights the uncertainties associated with PAH methodologies, which can vary significantly due to different measurement methods, such as Tanner-Whitehouse and Bayley-Pinneau (24). In the Global IGFD Registry, PAH is not derived from a standardized methodology but is instead based on the investigator's choice. It should also be noted that reporting of bone age at rhIGF-1 initiation is lacking in the majority of patients (19). Nevertheless, understanding the factors that may predict height gain and improved NAH is important for clinicians when determining the best treatment strategies for their patients. Based on multivariate analyses, naivety from all growth-promoting treatments (rhGH, rhIGF-1, and steroids) and biological mother height were predictive factors of HtSDS gain to NAH in the overall NAH population receiving rhIGF-1 therapy in the present study. For NPP patients, lower baseline HtSDS, higher PAH, and higher biological mother height were predictive of HtSDS gain to NAH. Lower baseline HtSDS, and thus a more severe height phenotype, was previously shown to be a predictor of greater change in HtSDS in the first year of rhIGF-1 treatment in NPP patients (15). Starting rhIGF-1 treatment at a younger age was also reported to be a predictor of better response to rhIGF-1 (15). In this analysis, NPP patients who started rhIGF-1 treatment at ≤10 years of age had a numerically greater cumulative ΔHtSDS at each year of treatment than patients who started rhIGF-1 treatment above 10 years, supporting the previous conclusion that starting rhIGF-1 at a younger age leads to improved height outcomes. Unlike catch-up growth observed in children treated with rhGH for approved disorders (25), it is important to note that height gain in the first year of rhIGF-1 treatment was not predictive of HtSDS gain to NAH in the current study. Catch-up growth on rhGH and rhIGF-1 therapy differs significantly because GH has IGF-I-independent effects on linear growth, which limits the ability to fully compensate for growth defects after rhIGF-1 treatment in individuals with complete GH insensitivity (15, 17). This explains why patients with Laron syndrome, the most severe form of GH insensitivity, do not achieve NAH close to the normal range after rhIGF-1 therapy, unlike NPP patients who have a milder phenotype and experience greater height gains (15, 17). Conversely, rhGH treatment in patients with severe GH deficiency can often enable complete catch-up growth, resulting in NAH close to the normal range. Patients with less severe GH deficiency or other indications are less likely to reach NAH within the normal range (15, 26).

It should be noted that some patients terminated rhIGF-1 treatment prior to reaching NAH for reasons other than achieving AH, so the full potential effectiveness of rhIGF-1 treatment may not have been observed. In the overall population, the next most frequently reported reason for treatment discontinuation after achieving AH was lack of effectiveness. It is worth noting that there may have been higher expectations for rhIGF-1 treatment effectiveness than is realistic, which may have led to patients withdrawing from treatment prematurely.

Safety data reported in the NAH population in this analysis aligned with those observed in the overall registry population, were consistent with the previously reported long-term safety profile of rhIGF-1, and resulted in treatment discontinuation in a minority of cases (14, 17, 27). The proportion of patients who reported a hypoglycemic event, a known side effect of rhIGF-1 treatment, was similar to previous reports from the Eu-IGFD Registry and clinical studies (18–49%) (10, 14, 15, 17, 27). Interestingly, our analysis suggested that the number of hypoglycemic events was highest in the first year of rhIGF-1 treatment, which has not been previously reported. It should be noted that the inclusion of patients with previous exposure to IGF-1 therapy in this analysis means the first year of rhIGF-1 treatment recorded as part of the Global IGFD Registry does not reflect the first year of IGF-1 treatment overall. It is therefore not possible to determine a clear relationship between the length of rhIGF-1 treatment and hypoglycemic event rate from the present study.

Rates of neoplasia in the NAH population aligned with the previous report from the Eu-IGFD Registry (15). As previously suggested, clinicians should be vigilant of any symptoms of malignancy and only prescribe rhIGF-1 within the approved label (11). In addition to hypoglycemia and neoplasia, other AEs such as headache, papilledema, and acromegalic features were observed. Headache and papilledema can suggest increased intracranial pressure and warrant close monitoring during treatment (28). Acromegalic features should also be assessed regularly to manage potential long-term effects. These AEs underscore the need for vigilant clinical surveillance in patients receiving rhIGF-1 treatment. There were no new concerns about the frequency or nature of AEs in children with severe growth failure receiving rhIGF-1 treatment over the long term in this analysis.

### Limitations of This Study

Diagnosis of SPIGFD for the Global IGFD Registry is determined at the discretion of the reporting physician based on height assessment using country-specific growth charts and measurement of circulating IGF-I levels using local commercially available assays that may lack sensitivity and proper controls to establish published normative values (1, 9). This, alongside a lack of standardized genetic testing, complicates the diagnosis of SPIGFD, which could result in some patients being inappropriately included or excluded from the Global IGFD Registry (9). Furthermore, as the Global IGFD Registry is an extension of the Eu-IGFD Registry, in which patients with and without SPIGFD were included, some patients included in the current analyses were not reported to have SPIGFD, which may have resulted in an overestimation of rhIGF-1 effectiveness. Meanwhile, the low number of patients in each population in later years of follow-up may have skewed the annualized height velocity and ΔHtSDS data, so these data should be interpreted with caution. The small number of NPP patients with Laron syndrome in this study limited the ability to conduct a specific analysis in this subgroup. Additionally, the high proportion of non-NPP patients in the Laron syndrome cohort may have led to an underestimation of rhIGF-1's effectiveness in this population. Although most patients in this study discontinued treatment after reaching AH, the inclusion of patients who discontinued treatment for reasons other than achieving AH may also have resulted in an underestimation of overall rhIGF-1 effectiveness. Finally, quality of life data were not described as part of this analysis as data were available for too few patients, preventing an assessment of the impact of rhIGF-1 treatment on patient well-being for those who reached NAH. Despite these limitations, this analysis provides valuable insight into the long-term effectiveness and safety of rhIGF-1 treatment in a relatively large cohort of patients who achieved NAH in the real-world setting.

## Conclusion

Data from this larger cohort of patients with severe growth failure who achieved NAH during or after rhIGF-1 treatment confirmed the effectiveness and safety of rhIGF-1 observed in clinical trials and demonstrated that these findings are applicable in a real-world setting. In this study, patients who were treatment-naïve and prepubertal at the start of rhIGF-1 treatment showed the greatest improvements in NAH. Additionally, a lower baseline HtSDS, indicating a more severe height phenotype, was predictive of greater height gain to NAH in these patients. Together, these data indicate that rhIGF-1 therapy may be more effective at enabling patients with severe growth failure due to SPIGFD to achieve NAH when rhIGF-1 treatment is started at the prepubertal stage in patients with no prior exposure to growth-promoting treatments.

## Data Availability

Qualified researchers may request access to patient-level study data that underlie the results reported in this publication. Additional relevant study documents, including the clinical study report, study protocol with any amendments, annotated case report form, statistical analysis plan, and dataset specifications may also be made available. Patient-level data will be anonymized, and study documents will be redacted to protect the privacy of study participants. Where applicable, data from eligible studies are available 6 months after the studied medicine and indication have been approved in the United States and European Union or after the primary manuscript describing the results has been accepted for publication, whichever is later. Further details on Ipsen's sharing criteria, eligible studies, and process for sharing are available at https://vivli.org/members/ourmembers/. Any requests should be submitted to www.vivli.org for assessment by an independent scientific review board.
